# Italian translation, cultural adaptation and validation of the PedsQL™ 3.0 Diabetes Module questionnaire in children with type 1 diabetes and their parents

**DOI:** 10.1186/s12955-014-0115-2

**Published:** 2014-07-19

**Authors:** Giuseppe d’Annunzio, Sara Gialetti, Chiara Carducci, Ivana Rabbone, Donatella Lo Presti, Sonia Toni, Eugenio Zito, Sara Bolloli, Renata Lorini, Ornella Della Casa Alberighi

**Affiliations:** 1 Pediatric Clinic, IRCCS - Istituto Giannina Gaslini, Genoa, Italy; 2 Endocrinology-Diabetology Unit, Department of Pediatrics of the University and Hospital, Bambino Gesù Pediatric Hospital, Rome, Italy; 3 Endocrinology-Diabetology Unit, Department of Science of Public Health and Pediatric, University of Turin, Turin, Italy; 4 Pediatric Clinic Unit, AOU Policlinico Vittorio Emanuele, Catania, Italy; 5 Diabetologic Unit, Meyer’s Children Hospital, Florence, Italy; 6 Department of Pediatrics, Federico II University, Naples, Italy; 7 Clinical Pharmacology & Clinical Trial Unit, Scientific Direction, IRCCS - Istituto Giannina Gaslini, via G. Gaslini, 3-5, Genoa, 16147, Italy

**Keywords:** Pediatric Quality of Life, PedsQL™3.0 Diabetes Module questionnaire, International validation, Italian

## Abstract

**Background:**

The PedsQL™3.0 Diabetes Module is a widely used instrument to measure the disease-specific health-related quality of life summary measures in children and adolescents with type 1 diabetes. After cultural adaptation, we confirmed reliability and validity of PedsQL™3.0 Diabetes Module in its Italian version.

**Methods:**

Participants were 169 Italian children and adolescents with type 1 diabetes aged 5–18 years and 100 parents. Reliability was determined by internal consistency using Cronbach’s coefficient alpha, and test-retest reliability by intra-class correlation coefficient (ICC). Validity was assessed through factor validity examined by exploratory factor analysis, and discriminant validity examined through multitrait/multi-item scaling analysis. Discriminant validity with respect to dichotomous patients’ characteristics at baseline was also examined through a multivariate analysis on the summary measures using the Wilks’ Lambda test.

**Results:**

Data completeness was optimal. Item internal consistency was satisfied at 89% for the child self-report scales and at 100% for the parents’ proxy-report scales. Most diabetes module scales was acceptable for group comparisons. Discriminant validity was satisfied for 71% of children and adolescents and for 82% of parents. A ≥70% Cronbach’s α coefficient was found for the summary measures of both reports. For the test-retest reliability, the ICC coefficients ranged from 0.66 (i.e., the *Worry* scale) to 0.82 for the other scales of the child self-report. The ICC coefficients were ≥0.87 for all the parents’ proxy-report scales. Factor analysis showed that the PedsQL™3.0 Diabetes Module for child self-report could be summarized in 10 components, which explained the 62% of the variance. For the parent proxy-report the statistical analysis selected 9 factors, which explained about 68% of variance. The external discriminant validity of the PedsQL™3.0 Diabetes Module summary measures were compared across gender, age, time since diagnosis and HbA1c mean cut off values. Significant differences in the “*Treatment adherence*” scale and in the “*Communication*” scale were observed across age, and by time since diagnosis.

**Conclusions:**

The results show the reliability and validity of the Italian translation of the PedsQL™3.0 Diabetes Module, supporting therefore its use as an outcome measure for diabetes cross-national clinical trials and research.

## Background

In the last decade, quality of life (QOL) has emerged as an important health objective in diabetes management, being positively related to degree of metabolic control [[1],[2]]. According to the World Health Organization, health is a state of complete physical, mental and social well-being and not merely the absence of disease or infirmity [[3]]. Therefore, a health-related quality of life (HRQOL) measurement should include physical, mental and social health aspects, be based on different age patient’s perception, and provide evidence of acceptance and reliability. In the perspectives of the health care provider, HRQOL is increasingly becoming a recognized measure of treatment outcome based on the concept that an illness affects all domains of the patient’s and family’s life. Moreover, according to the FDA Guidelines [[4]], HRQOL measurement by means of validated questionnaires is an essential outcome in clinical research in different settings as in pediatrics for international observational studies and clinical trials [[5]].

Juvenile diabetes management involves young patients and all family members, and can interfere with familiar dynamics and habits, affecting both patients’ and parents’ HRQOL [[1],[2]]. The availability of parent-proxy report scales is essential to obtain complementary information in juvenile diabetes HRQOL matters. As a consequence, several generic and disease-specific instruments to measure HRQOL have been developed and validated in diabetes. The Pediatric Quality of Life Inventory (PedsQL™) is a modular instrument for measuring HRQOL in children and adolescents ages 2 to 18 [[6]]. The PedsQL™4.0 Generic Core Scales are multidimensional child self-report and parent proxy-report scales developed as the generic core measure to be integrated with the PedsQL™ Disease-Specific Modules into a one-measurement system. The PedsQL™4.0 Generic Score Scales distinguish between healthy children and those affected by acute or chronic diseases and provide evidence of good feasibility, reliability and sensitivity to different diseases in several reports [[7]–[9]]. The PedsQL™3.0 Diabetes Module questionnaire has been assessed to measure diabetes-specific QOL dimensions in both children and their parents: either PedsQL™4.0 or PedsQL™3.0 Diabetes Module have been tailored for a broad age range including child self-report for ages 5–18 years, and parents’ proxy-report for their children for ages 2–18 years [[10]]. Recently, feasibility, reliability and validity of the new electronic version of e-PedsQL™ for type 1 and type 2 diabetes has been successfully demonstrated [[11]].

Due to the complex construction of each instrument, these measures cannot be assumed to be invariant to cultural diversity, thus requiring cultural adaptation [[12]], and verification of their psychometric properties [[13]–[15]]. The PedsQL™3.0 Diabetes Module questionnaire has already been translated and validated for many languages although not yet in Italian (see the listing of existing translations available from http://www.pedsql.org/PedsQL-Translation-Tables.doc. Accessed February 18, 2014).

The purpose of the present study was the translation, its cultural adaptation and statistical validation of the Italian version of the PedsQL™3.0 Diabetes Module questionnaire for a broad age range including children’s and adolescents’ self-reports for ages 5–18 years, and parents’ proxy-reports for their children for ages 5–18 years from six Italian pediatric centers for diabetes.

## Methods

### The questionnaire

The PedsQL™3.0 Diabetes Module includes 28 items distributed into 5 scales: 1) *Diabetes symptoms* (11 items); 2) *Treatments barriers* (4 items); 3) *Treatment adherence* (7 items); 4) *Worry* (3 items); 5) *Communications* (3 items). The child instrument differs by age group: 5 to 7, 8 to 12 and 13 to 18 years. The parent’s version also differs by child’s age group: 2 to 4, 5 to 7, 8 to 12 and 13 to 18 years. The items for each of the forms are essentially identical, differing in developmentally appropriate language, or first-person or third-person tenses.

The goals of the translation and cultural validation of the questionnaire text contents were to develop a version of the PedsQL™3.0 Diabetes Module that sounded natural in Italian, conceptually equivalent to the original US English version, and easy to understand and to answer for Italian children with type 1 diabetes. Translation and cultural validation were performed according to the guidelines provided by the MAPI Research Trust (at http://www.pedsql.org/PedsQL-Linguistic-Validation-Guidelines.doc). After minor modifications of the local translation, the Italian final version of the PedsQL™3.0 Diabetes Module was produced. All translation procedures were reported to the MAPI Research Trust, to rate the equivalence between the final Italian version and the original US English version*.*

### Study population

The eligible patients were on intensified basal-bolus insulin therapy or on continuous subcutaneous insulin infusion. No Italian-speaking subjects or those with linguistic problems, and those with any comorbidity that would have affected outcomes or care (i.e., chromosomopathy, and neurological impairment) were excluded. The psychologist SG of the coordinating center provided training material (i.e., written standardized procedures and instructions via conference calls) to colleagues at the participating centers. Written informed assents and consents were obtained by minors aged ≥12 years and parents, respectively, prior to study entry. The questionnaires were randomly administered to children and parents during follow-up visits at the six participating centers with proved experience in pediatric type 1 diabetes management and belonging to the Italian Society of Pediatric Endocrinology and Diabetes (ISPED) (Catania, Florence, Genoa, Naples, Rome, Turin). Patients and parents completed the questionnaires separately. The Ethical Committees of all centers approved this study.

### Psychometric evaluation and statistical analyses

The evaluation of the psychometric properties of the Italian version of the questionnaire involved the following steps:

1. *Scoring of the PedsQL™3.0 Diabetes Module according to the scoring manuals*. For each item the 5-point response scale (0 = never a problem; 1 = almost never a problem; 2 = sometimes a problem; 3 = often a problem; 4 = almost always a problem) was reversed and linearly transformed in a 0–100 scale where 0 = 100, 1 = 75, 2 = 50, 3 = 25, and 4 = 0. Therefore, higher scores indicate better QOL. For each questionnaire scale, the score was summed up and divided by the number of items answered.

2. *Evaluation of completeness at item and scale levels*.

3. *Calculation of the scale level descriptive statistics,* i.e*.* mean and 95% confidence intervals (CI), and percentage of ceiling and floor.

4. *Evaluation of internal consistency or reliability* – i.e., the Cronbach’s α coefficient (α = k x r /[1 + (k - 1) x r]; with k = number of items and r = mean correlation) [[16]], and the Squared Multiple Correlation (SMC) index, which were calculated for each item and for each scale.

5. *Evaluation of the multitrait/multi-item correlation matrix* to assess the items’ internal consistency by means of the Pearson correlation coefficient; the equality of item-scale correlations; the items’ discriminant validity.

6. *Evaluation of the test-retest correlation for temporal stability* in a two-week interval by means of the intra-class correlation (ICC) coefficient.

7. *Evaluation of the aggregating dimensions of the PedsQL™3.0 Diabetes Module scales* by means of a factor analysis (principal components, oblique rotation – i.e., oblimin).

8. *Evaluation of the discriminant validity of the questionnaire* with respect to the dichotomous patients’ characteristics (sex, time since diagnosis ≤1 year or >1 year, mean value of HbA1c ≤7.5 mg/dL or >7.5 mg/dL) by performing the Student’s t test and the 95% CI of the mean difference for each scale and a multivariate analysis on the summary measures using the Wilks’ Lambda test. Discrimination ability of the questionnaire with respect to patient ages according to the questionnaires (5–7 years, 8–12 and 13–18 years) was analysed for each scale using an ANalysis Of VAriance (ANOVA), and the appropriate multiple comparisons between groups. The 95% CI of each group’s means and of the between-group differences were calculated. A multivariate analysis on the summary measures was also performed using the Wilks’ Lambda test.

The BMDP statistical software (University of California Press, Release 2009 - Berkeley, Los Angeles, Oxford) was used for computation.

### Results study population

Between November 2011 to June 2012, 172 Italian children and adolescents with type 1 diabetes aged 5–18 years and 104 parents were recruited. The statistical analysis was performed on data from questionnaires of 169 children and 100 parents, having excluded three patients and four parents who did not satisfy an eligibility criterion (child’s time since diabetes diagnosis ≥ 3 months). Participants’ characteristics are reported in Table 1.

**Table 1:** Participants’ characteristics

	**Catania**	**Florence**	**Genoa**	**Naples**	**Rome**	**Turin**	**Totals**
**Children-** male, N (%)	8 (67%)	18 (51%)	35 (57%)	9 (56%)	11 (34%)	9 (69%)	90 (53%)
Age, 5–7 yers, N (%)	11 (92%)	5 (14%)	5 (8%)	7 (44%)	5 (16%)	11 (85%)	44 (26%)
Age, 8–12 years, N (%)	1 (8%)	25 (71%)	22 (36%)	7 (44%)	24 (75%)	2 (15%)	81 (48%)
Age, 19–18 years, N (%)	n.a.	5 (14%)	34 (56%)	2 (13%)	3 (9%)	n.a.	44 (26%)
HbA1c ≤ 7.5%, N (%)	6 (50%)	17 (49%)	17 (28%)	2 (13%)	17 (53%)	6 (46%)	65 (38%)
HbA1c ≤ 7.5%, N (%)	6 (50%)	18 (51%)	44 (72%)	14 (88%)	15 (47%)	7 (54%)	104 (62%)
Type 1 diabetes duration (≥3 months - <1 year), N (%)	1 (8%)	3 (9%)	2 (3%)	n.a.	5 (16%)	2 (15%)	13 (8%)
Type 1 diabetes duration (>1 year), N (%)	11 (92%)	32 (91%)	59 (97%)	16 (100%)	27 (85%)	11 (85%)	156 (92%)
**Parents**							
Mothers, N (%)	n.a.	n.a.	23 (60.5%)	12 (75%)	13 (65%)	7 (54%)	55 (55%)
Fathers, N (%)	n.a.	9 (69%)	8 (21%)	4 (25%)	1 (5%)	2 (15%)	24 (24%)
Both, N (%)	n.a.	4 (31%)	7 (18%)	n.a.	6 (30%)	4 (31%)	21 (21%)

HbA1c: mean values of glycosylated haemoglobin observed during the previous year; N.: number; n.a.: not available.

### Translation and cultural validation

The nomenclature of the scales did not need any modification. Comparing the back-translations with original versions, 13 items were rephrased by the translators, together with the psychologist SG and the study principal investigator. During the test, the psychologist verified the due intelligibility of the items in the questionnaire: two items were rephrased with the help of patients, parents and the psychologist to increase the clarity.

### Psychometric evaluation and statistical analysis

Completeness was optimal both at the item and scale level (i.e., the percentage of missing item responses was 0.04 in both children and parents), with almost 100% of items answered and scales completed. Table 2 shows the mean and 95% CI for the scales belonging to child self- report and parents’ proxy-report scales. The means of each scale were quite similar, except for the “*Treatment barriers*” scale for which parents’ proxy-report scale showed a significant lower score. A Cronbach’s α coefficient <70% was recorded for all the items in the child self-report scale (range 0.62 - 0.67), and for the “*Diabetes symptoms*”, “*Treatment barriers*” and “*Communication*” of parents’ proxy-report scales (Table 2). The proportion of patients with a floor effect ranged from 0% to 3% in the child self-report scales, and from 0% to 4% in the parent proxy-report scales. The proportion of patients with a ceiling effect ranged from 0.6% to 24% in the child self-report scales and from 2% to 23% in the parents’ proxy-report scales. The Pearson’s correlation coefficient for the item internal consistency was satisfied at 89% and 100% for the child self-report and parents’ proxy-report scales, respectively. Discriminant validity was satisfied for 71% and 82% of items. Internal consistency was adequate with a Cronbach’s α coefficient ≥70% for the total scores of both reports. There is no evidence of item overlap by the evaluation of the multitrait/multi-item correlation matrix to assess the items’ internal consistency. The inter-correlations between the child self-report and parents’ proxy-report scales ranged from 0.28 to 0.54, being most of them in the medium effect size range. “*Diabetes symptoms*” and “*Treatments barriers*” scales had the strongest correlations. For test-retest reliability, a subset of patients (n = 94) and parents (n = 17) completed the PedsQL™3.0 Diabetes Module during a routinely scheduled clinic visit in approximately 10 minutes after maximum two weeks since the first questionnaire administration. The ICC ranged from 0.66 to 0.82 for all items of the child self-report scales, the “*Worry*” scale only having a value <70%. For the parents’ proxy-report scales, the ICC ranged from 0.87 to 0.99 for all items (Table 3).

**Table 2:** Scale descriptive and internal consistency for PedsQL™ 3.0 Diabetes Module child self-reports and parent proxy-reports

			**95%C.I.**							
**Subscale**	**N. of items**	**N.**	**Mean**	**Lower limit**	**Upper limit**	**S.D.**	**Min.**	**Max.**	**SMC**	**Cronbach’s α**
**Child self-report**										
**Diabetes self symptoms**	11	169	64.69	62.31	67.06	15.65	9.09	100	0.28	0.61
**Treatment barriers**	4	169	79.22	77.11	81.32	20.02	0.00	100	0.23	0.64
**Treatment adherence**	7	169	82.35	80.25	84.46	13.89	42.86	100	0.20	0.65
**Worry**	3	169	66.91	63.47	70.36	22.70	0.00	100	0.18	0.67
**Communicationn**	3	169	73.42	69.64	77.21	24.93	0.00	100	0.20	0.65
**Total score**	28	169	73.32	71.33	75.30	13.07	20.79	97.24		0.69
**Parent proxy report**										
**Diabetes symptoms**	11	100	67.75	64.59	70.91	15.92	20.46	100	0.4146	0.65
**Treatment barriers**	4	100	71.19	67.21	75.14	20.06	0	100	0.5107	0.64
**Treatment adherence**	7	100	80.03	77.24	82.82	14.05	28.57	100	0.2368	0.71
**Worry**	3	100	66.58	61.43	71.74	25.99	0	100	0.0976	0.78
**Communication**	3	100	74.25	96.57	78.93	23.60	0	100	0.3748	0.69
**Total score**	28	100	71.96	96.21	74.71	13.87	27.19	95.68		0.74

N.: number of participants; S.D.: standard deviation; SMC: Squared Multiple Correlation (Index); 95% C.I.: 95% confidence intervals.

**Table 3:** Test-retest reliability ICCs for child self-reports* and parent proxy-reports*

**Item**	**ICC child (N = 94)**	**ICC proxy (N = 17)**
**Diabetes symptoms**		
1	0.6805	0.9356
2	0.6315	0.5949
3	0.6300	0.7813
4	0.8094	0.9189
5	0.7484	0.8762
6	0.4713	0.9149
7	0.7110	0.9061
8	0.6427	0.8851
9	0.6228	0.9422
10	0.7879	0.9829
11	0.5926	0.8005
Total	0.8085	0.9241
**Treatment barriers**		
1	0.7226	0.9380
2	0.8704	0.9836
3	0.7667	0.9623
4	0.5582	0.9866
Total	0.8234	0.9868
**Treatment adherence**		
1	0.7302	0.8431
2	0.7510	0.9223
3	0.7317	0.3077
4	0.5639	0.6712
5	0.4828	0.9075
6	0.2940	0.3334
7	0.6384	0.8863
Total	0.8079	0.8656
**Worry**		
1	0.6922	0.9708
2	0.5740	0.8815
3	0.5067	0.8699
Total	0.6598	0.9346
**Communication**		
1	0.6060	0.6842
2	0.6455	0.8972
3	0.7157	0.9478
Total	0.7506	0.8858

*Reported for ages 5 years and older.

**Figure 1:**
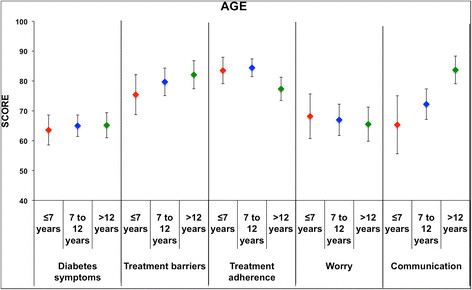
**Discrimination ability of the questionnaire with respect to grouped ages of patients.** Mean summary scores for three groups of children (i.e., 5–7 year-, 8–12 year-, and 13–18 year-old subjects) are compared. Significant differences in the “*Treatment adherence*” and the “*Communication*” scales were observed in the 13–18 year-old group.

**Figure 2:**
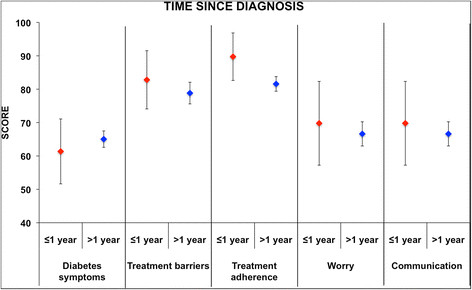
**Discrimination ability of the questionnaire with respect to time since diagnosis.** Mean summary scores for two groups of patients (i.e., either time since diagnosis ≤1 year or >1 year) are compared. A significant difference in the “*Treatment adherence*” scale was observed.

Factor analysis showed that the PedsQL™3.0 Diabetes Module for child self-report could be summarized into 10 components, which explain the 62% of the variance. The “*Diabetes symptoms*” items were split into 5 different factors, “*Treatment barriers*” into 2, “*Treatment adherence*” into 3, “*Communication*” into 2, while “*Worry*” was represented by one factor only (Table 4). For the parent proxy-reports, the statistical analysis selected 9 factors, which explained about 68% of variance; “*Diabetes symptoms*”, “*Treatment barriers*” and “*Treatment adherence*” were split into 4 factors, “*Communication*” into 2, and “*Worry*” was the only measure described by only one factor (Table 5). To assess external discriminant validity of PedsQL™3.0 Diabetes Module, the “*Diabetes symptoms*”, “*Treatments barriers*”, “*Treatment adherence*”, “*Worry*” and “*Communication*” summary measures were compared across gender, age, time since diagnosis and HbA1c mean cut off values. Results are summarized in Figures 1 and 2. Differences in “*Treatment adherence*” were observed by age group: the mean value of children aged >12 years was statistically different when compared to that of group aged ≤7 years (p < 0.05) and group aged >7 years and ≤12 year (p < 0.01). Differences in “*Communication*” were observed by age: the mean value of children aged >12 years was statistically different in comparison with that of the group aged ≤7 years (p < 0.01) and the group aged >7 and ≤12 years (p < 0.05). Differences in “*Treatment adherence*” were observed for time since diagnosis: the mean value of the group diagnosed ≤1 year was statistically different when compared to that with >1 year since diagnosis (p < 0.05); lower scores were observed for the group with >1 year since diagnosis.

**Table 4:** PedsQL™ 3.0 diabetes module questionnaire factor loadings for child self-reports*

**Item**	**Factor 1**	**Factor 2**	**Factor 3**	**Factor 4**	**Factor 5**	**Factor 6**	**Factor 7**	**Factor 8**	**Factor 9**	**Factor 10**
**Diabetes symptoms**										
1	0.07	0.081	0.055	**0.652**	0.173	0.089	−0.216	0.013	−0.103	0.06
2	−0.136	0.191	−0.048	0.149	**0.576**	−0.031	−0.052	−0.219	0.06	0.207
3	0.099	0.152	0.046	−0.114	**0.439**	0.321	−0.005	−0.169	−0.078	0.327
4	0.047	−0.049	0.073	0.056	−0.038	0.097	−0.032	0.005	−0.11	**0.811**
5	0.115	0.149	−0.154	0.02	0.089	0.117	0.013	**0.666**	−0.005	0.037
6	−0.118	**0.745**	0.072	−0.008	−0.033	0.078	−0.059	0.326	−0.134	−0.155
7	−0.021	**0.368**	0.078	0.291	0.221	−0.101	−0.028	0.283	−0.06	0.174
8	0.084	**0.744**	−0.052	−0.089	−0.055	−0.015	0.001	−0.097	0.33	0.065
9	0.149	**0.603**	−0.061	0.368	−0.004	0.01	0.211	−0.206	0.067	0.077
10	0.201	−0.037	0.052	0.032	−0.041	−0.024	0.163	0.35	0.086	**0.391**
11	0.23	0.053	−0.04	**0.435**	0.01	0.068	0.052	0.099	0.241	0.235
**Treatment barriers**										
1	0.049	0.156	**0.504**	−0.191	−0.197	0.079	−0.159	0.384	0.132	0.058
2	−0.012	−0.105	**0.796**	−0.01	−0.039	−0.011	−0.09	−0.175	0.124	0.231
3	0.218	−0.156	0.131	0.335	−0.037	0.136	0.095	0.015	**0.544**	−0.135
4	−0.008	−0.034	**0.547**	0.355	0.008	0.005	0.143	0.246	0.269	−0.182
**Treatment adherence**										
1	0.015	0.169	0.084	−0.133	0.115	0.006	−0.004	−0.036	**0.733**	−0.061
2	0.315	0.005	0.123	−0.478	**0.493**	0.093	0.005	0.169	0.046	−0.011
3	−0.174	0.084	−0.016	0.037	0.169	0.038	**0.531**	0.156	0.254	0.239
4	0.131	0.032	0.429	0.371	0.066	0.019	**0.432**	−0.025	−0.299	−0.09
5	−0.017	−0.025	−0.075	−0.094	−0.091	0.117	0.79	**−0.133**	−0.008	−0.111
6	0.027	−0.165	−0.047	0.063	**0.735**	−0.02	0.099	0.136	0.075	−0.155
7	−0.046	0.016	0.073	−0.164	0.201	−0.143	**0.462**	0.293	−0.038	0.162
**Worry**										
1	0.274	0.061	−0.154	0.039	−0.03	**0.656**	0.13	0.016	−0.144	−0.062
2	−0.176	0.001	0.039	−0.089	−0.085	**0.778**	0.032	−0.011	0.081	0.16
3	−0.122	−0.108	0.184	0.253	0.229	**0.575**	−0.126	0.179	0.156	−0.078
**Communication**										
1	**0.795**	−0.009	0.057	0.075	−0.141	−0.007	−0.042	0.005	0.047	0.054
2	**0.867**	−0.004	−0.023	−0.044	0.121	−0.044	−0.068	0.056	0.023	0.039
3	0.263	0.244	**0.567**	−0.075	0.145	0.098	0.121	−0.315	−0.135	0.135

*Reported for ages 5 years and older.

PedsQL™3.0 for child self-reports could be summarized into 10 components, which explain the 62% of the variance. *Eingenvalue* cut off value: 1.0; bold = highest factor loading for each item.

**Table 5:** PedsQL™ 3.0 Diabetes Module questionnaire Factor Loadings for parent proxy-reports*

**Item**	**Factor 1**	**Factor 2**	**Factor 3**	**Factor 4**	**Factor 5**	**Factor 6**	**Factor 7**	**Factor 8**	**Factor 9**
**Diabetes symptoms**									
1	0.402	0.278	**0.529**	−0.155	−0.042	0.045	0.107	0.042	0.181
2	0.209	0.052	**0.83**	0.175	−0.043	−0.025	0.089	0.007	−0.186
3	0.035	0.079	**0.838**	0.091	0.186	0.153	0.155	0.077	0.054
4	0.074	0.151	0.276	0.283	0.12	0.055	−0.111	**0.719**	0.147
5	0.397	0.325	0.149	−0.178	−0.098	0.129	−0.022	**0.584**	0.215
6	**0.76**	−0.032	−0.088	0.221	0.045	−0.046	0.143	0.2	−0.111
7	**0.681**	0.183	0.158	0.045	0.166	−0.039	0.254	0.065	0.057
8	0.466	0.154	0.012	0.172	0.07	0.333	0.021	0.128	**0.562**
9	**0.675**	0.015	0.269	0.085	0.153	0.123	−0.21	−0.107	0.044
10	**0.434**	0.386	0.188	0.023	0.02	0.108	0.042	0.016	−0.007
11	**0.449**	0.256	0.34	0.04	0.107	0.051	0.295	0.124	0.081
**Treatment barriers**									
1	0.215	0.085	0.192	**0.635**	0.17	−0.041	0.199	0.049	0.425
2	0.031	0.1	0.168	0.11	−0.01	0.114	**0.86**	0.097	0.09
3	0.081	**0.638**	0.293	−0.056	0.034	0.192	0.176	0	0.205
4	0.112	**0.524**	0.237	−0.125	0.103	0.404	0.341	0.032	−0.05
**Treatment adherence**									
1	−0.035	0.231	0.094	**0.735**	0.055	0.171	−0.124	0.006	−0.188
2	0.239	0.013	0.008	**0.772**	0.06	0.107	0.184	0.012	0.134
3	−0.014	0.207	0.024	0.013	0.222	**0.664**	0.063	0.074	0.079
4	0.317	0.318	0.198	−0.016	0.013	0.325	0.003	0.136	**−0.526**
5	0.026	−0.006	−0.174	−0.069	0.145	0.148	0.255	**0.688**	−0.239
6	−0.13	−0.073	0.044	0.348	−0.076	**0.629**	0.05	0.268	−0.138
7	0.16	−0.033	0.059	0.055	−0.115	**0.77**	0.008	−0.018	0.011
**Worry**									
1	0.396	0.213	−0.047	0.198	**0.674**	−0.023	−0.069	0.015	0.094
2	0.055	−0.003	0.03	0.038	**0.876**	0.087	0.053	−0.045	0.019
3	0.039	−0.104	0.126	−0.007	**0.8**	−0.041	0.093	0.201	−0.036
**Communication**									
1	0.081	**0.697**	0.021	0.454	−0.126	0	0.099	0.069	0.043
2	0.134	**0.784**	−0.075	0.217	0.033	−0.106	0.079	0.202	−0.187
3	0.252	0.393	0.139	0.091	0.162	−0.017	**0.649**	−0.041	−0.103

*Reported for ages 5 years and older.

PedsQL™3.0 for parent proxy-reports could be summarized into 9 components, which explain the 68% of the variance. *Eingenvalue* cut off value: 1.0; bold = highest factor loading for each item.

## Discussion

The results of the current study showed the reliability and validity of the PedsQL™3.0 Diabetes Module questionnaire in its Italian version after cultural adaptation of the disease-specific HRQOL summary measures, i.e., “*Diabetes symptoms*”, “*Treatments barriers*”, “*Treatment adherence*”, “*Worry*” and “*Communication*” in children and adolescents with type 1 diabetes. After an independent forward and backward translation of the validated US English version, the Italian version of PedsQL™3.0 Diabetes Module was tested in 169 Italian children and adolescents with type 1 diabetes aged 5–18 years and 100 parents by trained psychologists in the context of a multicenter observational longitudinal study. Even if complex and time requiring, the translational step-wise algorithm of the validation procedure according to the MAPI Research Trust allowed us to produce an Italian version of PedsQL™3.0 Diabetes Module which sounded user-friendly and easy to understand, culturally adapted for the country where it will be used, and conceptually similar to the original US English version. The main strength of this study was the enrollment of a wide range of children and parents willing to participate from Italian Northern-to-Southern regions at the six ISPED centers with proved experience in pediatric type 1 diabetes management. Therefore, the validating population represented the general Italian population of pediatric patients with type 1 diabetes.

The study aimed at validating the PedsQL™3.0 Diabetes Module questionnaire for use in Italy, rather than describing the QOL of pediatric patients with type 1 diabetes. Thus, the use of convenience sample should not impair our conclusions. Still, further applications of this version of instrument, currently ongoing on larger Italian pediatric population (Study EudraCT No. 201001923710), is warranted to confirm our results.

The psychometric properties of the Italian version may be considered satisfying. The mean scores in the different scales were comparable to those of the original version [[10]], and so were the scales with relevant percentages of ceiling or floor according to the IQOLA project approach [[13]]. In term of feasibility, time required to complete questionnaires was around 10–15 minutes. Items of the PedsQL™3.0 Diabetes Module had minimal missing responses, suggesting that patients and parents were able to provide easily QOL data. Similar observation was reported by Varni who evaluated the PedsQL™ in type 1 and type 2 diabetes [[10]], and in the more recent electronic version [[11]]. The PedsQL™3.0 Diabetes Module item internal consistency (i.e., the percentage of items with Pearson item-scale correlation ≥40%) was satisfied at 89% for the child self-report scales and 100% for the parents’ proxy-report scales. Discriminant validity (i.e., the percentage of items with higher Pearson correlation with other scales than with its own scale) was satisfied at 71% for the child self-report scales and at 82% for the parents’ proxy-report scales, respectively. The internal consistency reliabilities of PedsQL™3.0 Diabetes Module self-report and proxy-report as summary measures satisfied the recommended minimum α-coefficient standard of 0.70 for group comparisons [[13]]. For the items of the child self-report scales, they all were in the 0.60-0.70 range, while for the parents’ proxy-report item scales, “*Diabetes symptoms*”, “*Treatment barriers*”, and “*Communication*” ranged between 0.64-0.69. Strong correlation between the child self-reports and parents’ proxy-reports was showed between the same scales, with the exception of “*Treatment adherence*”, the evaluation of diabetes symptoms than emotional symptoms being the reason for this objectivity and easiness. The number of test items, item interrelatedness and dimensionality affect the value of alpha [[17]]. Although Cronbach alpha represents the lower bound of the reliability of a measurement instrument, and is a conservative estimate of actual reliability [[18]], scales that did not approach or meet the 0.70 standard should be used only for descriptive analyses.

Test-retest reliability was performed in a two-week interval, which was deemed adequate [[19]], having assumed that fluctuations due to external factors, such as disease and treatment variables, were unlikely and not expected to influence the scale functioning. ICC values among the children ranged from good to excellent except for the “*Worry*” scale. The ICC values for the parents’ proxy-report were excellent, despite the returned 30-50% of retest questionnaires was not met.

Validity was also assessed through factor validity and clinical/external discriminant validity. A factor analysis (principal components, oblique rotation - oblimin) for determining the items’ scale structure of an instrument along with assessment of items’ conceptual clarity was performed on PedsQL™3.0 Diabetes Module for child self-reports and parent proxy-reports to verify aggregating dimensions. This exploratory multivariate technique allows data reduction, i.e., it reduces the number of variables in an analysis by describing linear combinations of the variables that contain most of the information with (possibly) meaningful interpretation. The coefficients estimated from the linear combinations are called factor loadings. Factors were selected if their *eingenvalue* was ≥ 1. The variance explained by each factor is the *eigenvalue* for that factor. Although the original US English version has a five factor structure [[10]], exploratory factor analysis identified 10 factors for child self-reports and 9 factors for parent proxy-reports in our Italian version, as detailed in Table 3. Our findings are consistent with those from studies reporting on the factor structure of the original US English version [[20],[21]], and thus support the psychometric properties of the Italian translation version of the measure. As already reported by Nansel [[20]], parallel parent and child scales did not emerge. The factor structure of the PedsQL™3.0 Diabetes Module did not support the original five-factor scales. As reported by other authors [[21]], the findings indicate that the module is most reliable when used as one scale rather than five sub-scales. The external discriminant validity of the PedsQL™3.0 Diabetes Module summary measures were compared across gender, age, time since diagnosis and HbA1c mean cut off values. Significant differences in the “*Treatment adherence*” scale and in the “*Communication*” scale were observed across ages and by time since diagnosis.

## Conclusions

This study showed the reliability and validity of the Italian version of the PedsQL™3.0 Diabetes Module questionnaire, that is easy to understand and reduces possible cultural biases to a minimum. Therefore, this module could be used as an outcome measure for diabetes cross-national clinical trials and research.

## Abbreviations

PedsQL: Pediatric quality of life

ICC: Intra-class correlation coefficient

HbA1c: Haemoglobin glycosylated

QOL: Quality of life

HRQOL: Health-related quality of life

FDA: Food and Drug Administration

ISPED: Italian Society of Pediatric Endocrinology and Diabetes

CI: Confidence intervals

SMC: Squared multiple correlation

ANOVA: ANalysis of variance

IQOLA: International Quality of Life Assessment

## Competing interests

Dr. S. Gialetti was supported by an Agenzia Italiana del Farmaco (AIFA) Fellowship by grant Number FARM8MR2J7, study code EudraCT Number: 201001923710.

Statistical support for this publication was provided by grant Number FARM8MR2J7 (Dr. F. Bravi).

## Authors’ contributions

Conception and design: ODCA, GdA, SG. Study coordination: SG, GdA, SB, RL. Acquisition of clinical data, analysis and interpretation of data: ODCA, SG, GdA, SB, RL, CC, IR, DP, ST, EZ. Drafting and writing of manuscript: ODCA, GdA, SG. Revision of manuscript: ODCA, GdA, SG, CC, IR, DP, ST, EZ. All the authors read and approved the final manuscript.

## References

[B1] Hoey H Aanstoot HJ Chiarelli F Daneman D Danne T Dorchy H Fitzgerald M Garandeau P Greene S Holl R Hougaard P Kaprio E Kocova M Lynggaard H Martul P Matsuura N McGee HM Mortensen HB Robertson K Schoenle E Sovik O Swift P Tsou RM Vanelli M Åman J Good metabolic control is associated with better quality of life in 2.101 adolescents with type 1 diabetes Diabetes Care 2001 24 1923 1928 10.2337/diacare.24.11.1923 11679458

[B2] Quality of-life measures in children and adults with type 1 diabetes Diabetes Care 2010 33 2175 2177 10.2337/dc10-0331 20696865 PMC2945155

[B3] Constitution of the World Health Organization: Basic Document 1948 World Health Org, Geneva

[B4] Guidance for Industry: Patient-Reported Outcome Measures: Use in Medical Product Development to Support Labeling Claims 2006 MD, Center for Drug Evaluation and Research, Food and Drug Administration, Rockville

[B5] Varni JW Burwinkle TM Lane MM Health-related quality of life measurement in pediatric clinical practice: an appraisal and precept for future research and application Health Qual Life Outcomes 2005 3 1 9 10.1186/1477-7525-3-34 15904527 PMC1156928

[B6] Varni JW Seid M Rode CA The PedsQL™: measurement model for the pediatric quality of life inventory Med Care 1999 37 126 139 http://www.pedsql.org/Available from http://www.pedsql.org/. Accessed 17 October 2013 10.1097/00005650-199902000-00003 10024117

[B7] Varni JW Seid M Kurtin PS PedsQL™4.0: reliability and validity of the Pediatric Quality of Life Inventory™ version 4.0 Generic Core Scales in healthy and patient populations Med Care 2001 39 800 812 10.1097/00005650-200108000-00006 11468499

[B8] Varni JW Seid M Knight TS Uzark K Szer IS The PedsQL™4.0 Generic Core Scales: sensitivity, responsiveness, and impact on clinical decision-making J Behav Med 2002 25 175 193 10.1023/A:1014836921812 11977437

[B9] Varni JW Limbers CA Burwinkle TM Parent proxy-report of their children’s health-related quality of life: an analysis of 13,878 parents’ reliability and validity across age subgroups using the PedsQL™ 4.0 Generic Core Scales Health Qual Life Outcomes 2007 5 2 10.1186/1477-7525-5-2 17201923 PMC1769359

[B10] Varni JW Burwinkle TM Jacobs JR Gottschalk M Kaufman F Jones KL The PedsQL™ in type 1 and type 2 diabetes: reliability and validity of the pediatric quality of life inventory™ generic core scales and type 1 diabetes module Diabetes Care 2003 26 631 637 10.2337/diacare.26.3.631 12610013

[B11] Varni JW Limbers CA Burwinkle TM Bryant WP Wilson DP The ePedsQL in type 1 and type 2 diabetes: feasibility, reliability, and validity of the Pediatric Quality of Life Inventory™ Internet administration Diabetes Care 2008 31 672 677 10.2337/dc07-2021 18184893

[B12] Bullinger M Alonso J Apolone G Leplège A Sullivan M Wood-Dauphinee S Aaronson N Bech P Fukurama S Kaasa S Ware JE Jr Translating health status questionnaires and evaluating their quality: the IQOLA project approach. International Quality of Life Assessment J Clin Epidemiol 1998 51 913 923 10.1016/S0895-4356(98)00082-1 9817108

[B13] Ware JE Jr Gandek B Methods for testing data quality, scaling assumptions and reliability: the IQOLA project approach. International Quality of Life Assessment J Clin Epidemiol 1998 51 945 952 10.1016/S0895-4356(98)00085-7 9817111

[B14] Kalyva E Malakonaki E Eiser C Mamoulakis D Health-related quality of life (HRQoL) of children with type 1 diabetes mellitus (T1DM): self and parental perceptions Pediatr Diabetes 2011 12 34 40 10.1111/j.1399-5448.2010.00653.x 20546163

[B15] Sand P Kljajić M Schaller J Forsander G The reliability of the Health Related Quality Of Life questionnaire PedsQL 3.0 Diabetes Module™ for Swedish children with type 1 diabetes Acta Paediatr 2012 101 8 e344 e349 10.1111/j.1651-2227.2012.02706.x 22519935

[B16] Cronbach LJ Coefficient alpha and the internal structure of tests Psycometrika 1951 16 297 334 10.1007/BF02310555

[B17] Cortina J What is coefficient alpha: an examination of theory and applications J App Psychol 1993 78 98 104 10.1037/0021-9010.78.1.98

[B18] Novick MR Lewis C Coefficient alpha and the reliability of composite measurements Psychometrika 1967 32 1 1 13 10.1007/BF02289400 5232569

[B19] Streiner DL Norman GR Health Measurement Scales: a Practical Guide to their Development and Use 2003 Oxford University Press, Oxford

[B20] Nansel TR Weisberg-Benchell J Wysocki T Laffel L Anderson B Quality of life in children with type 1 diabetes: a comparison of general and diabetes-specific measures and support for a unitary diabetes quality-of-life construct Diabet Med 2008 25 1316 1323 19046222 10.1111/j.1464-5491.2008.02574.x PMC2597420

[B21] Lawrence JM Yi-Frazier JP Black MH Anderson A Hood K Imperatore G Klingensmith GJ Naughton M Mayer-Davis EJ Seid M Demographic and clinical correlates of diabetes-related quality of life among youth with type 1 diabetes J Pediatr 2012 161 2 201 207 10.1016/j.jpeds.2012.01.016 22361221 PMC4503360

